# Leiomyoma in the Transverse Colon With Resection

**DOI:** 10.7759/cureus.15535

**Published:** 2021-06-08

**Authors:** James R Pellegrini, Jose R Russe, Rezwan Munshi, Rebecca Smoller, Nausheer Khan

**Affiliations:** 1 Internal Medicine, Nassau University Medical Center, East Meadow, USA; 2 Internal Medicine, New York Institute of Technology College of Osteopathic Medicine (NYITCOM), Glen Head, USA; 3 Gastroenterology, Nassau University Medical Center, East Meadow, USA

**Keywords:** leiomyoma, transverse colon, colonic resection

## Abstract

Benign proliferations of smooth muscle cells are known as leiomyomas; these proliferations can occur in the colon and are typically found incidentally. Colonic leiomyomas are very rare and are most commonly found in the descending or sigmoid colon. A 59-year-old Hispanic female presented to the gastroenterology clinic for surveillance colonoscopy. The biopsy showed a submucosal microscopic leiomyoma in the transverse colon. The treatment of choice for most colonic leiomyomas is surgical excision. This rare case favors the notion that endoscopic polypectomy may be superior to surgical excision, ultimately providing a less-invasive and less-costly procedure without complications or recurrence.

## Introduction

Colonic leiomyomas are benign proliferations of smooth muscle cells, arising most often from the muscularis mucosa or muscularis propria layer of the colon. They typically present as incidental findings on colonoscopy and are benign as they rarely exhibit cellular atypia or malignant potential [1]. Colonic leiomyomas are extremely rare and account for only 3% of all gastrointestinal leiomyomas [2]. True gastrointestinal (GI) leiomyomas commonly occur in the esophagus or stomach, with occasional rectal manifestations [1]. The descending and sigmoid colon are the most prevalent locations of its occurrence in the colon, usually surfacing as ancillary small intraluminal polyps [3]. The following is a rare case of colonic leiomyoma, as we found a 7mm sessile polyp in the transverse colon on surveillance colonoscopy. The biopsy showed a submucosal microscopic leiomyoma. Since colonic leiomyomas are predominantly located in the rectum and sigmoid colon, this case exhibits a rare finding of a recurrent leiomyoma found in the transverse colon.

## Case presentation

**Figure 1:**
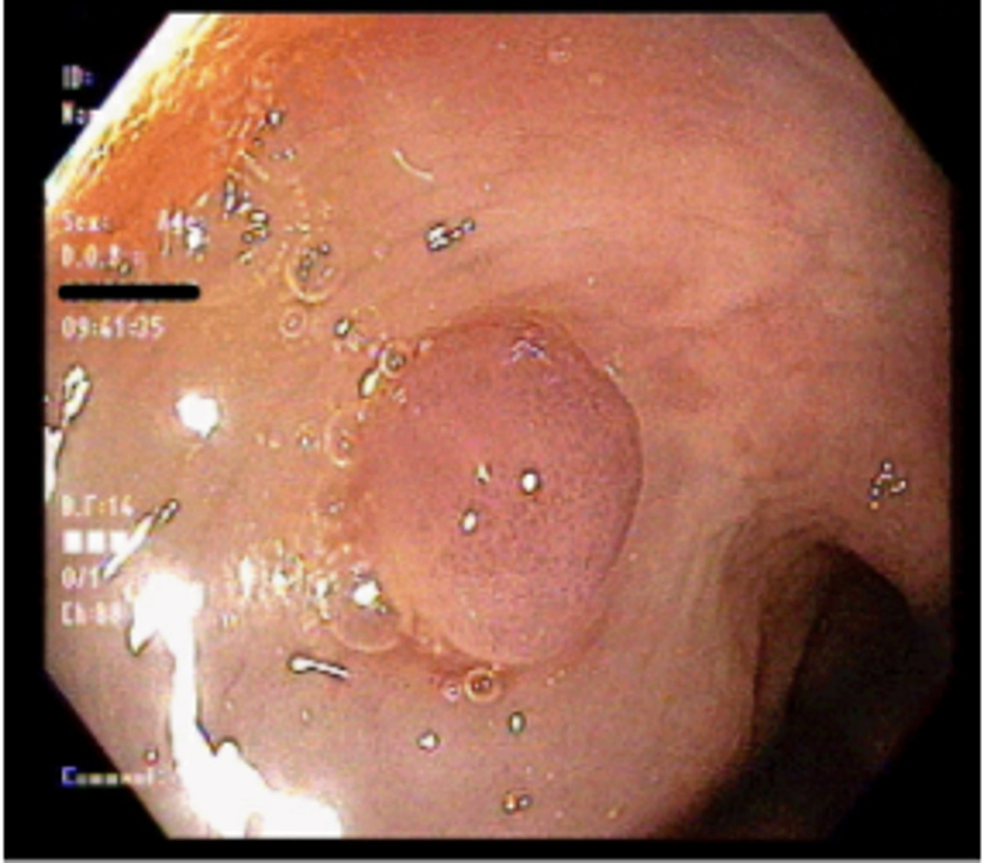
7mm sessile-appearing lesion

**Figure 2:**
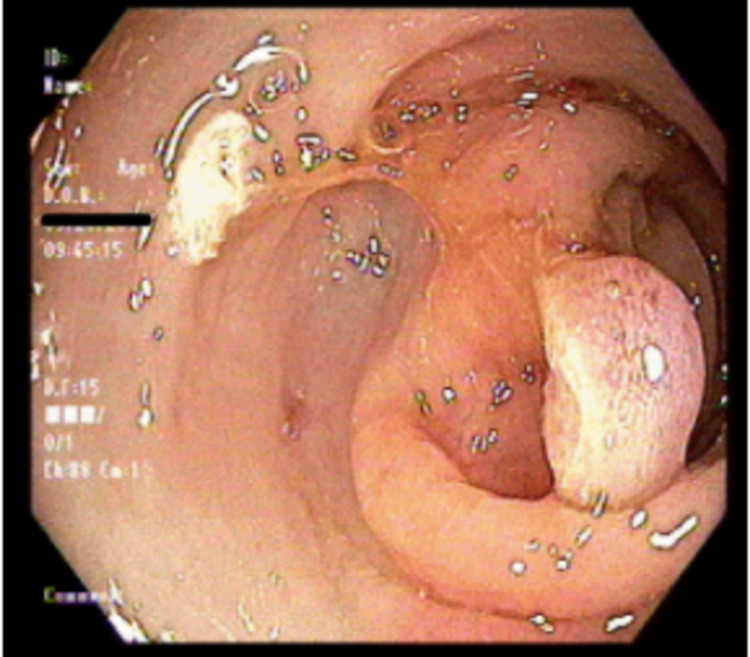
Resection of 7mm sessile-appearing lesion

**Figure 3:**
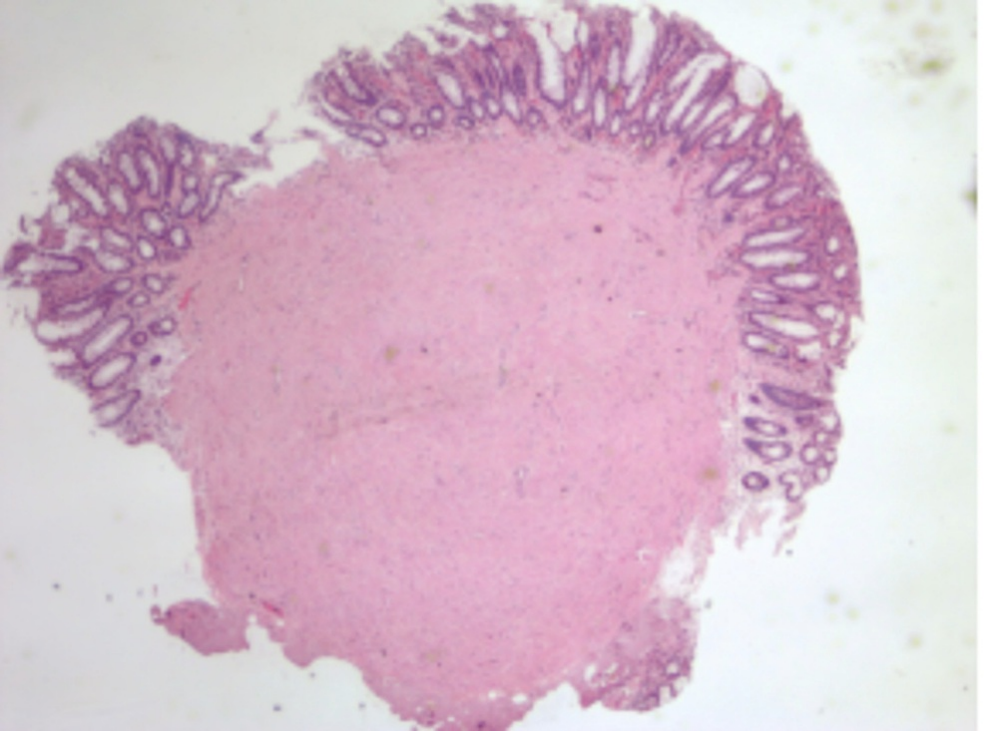
The image demonstrates a nodule underlying colonic columnar epithelium (20x)

**Figure 4:**
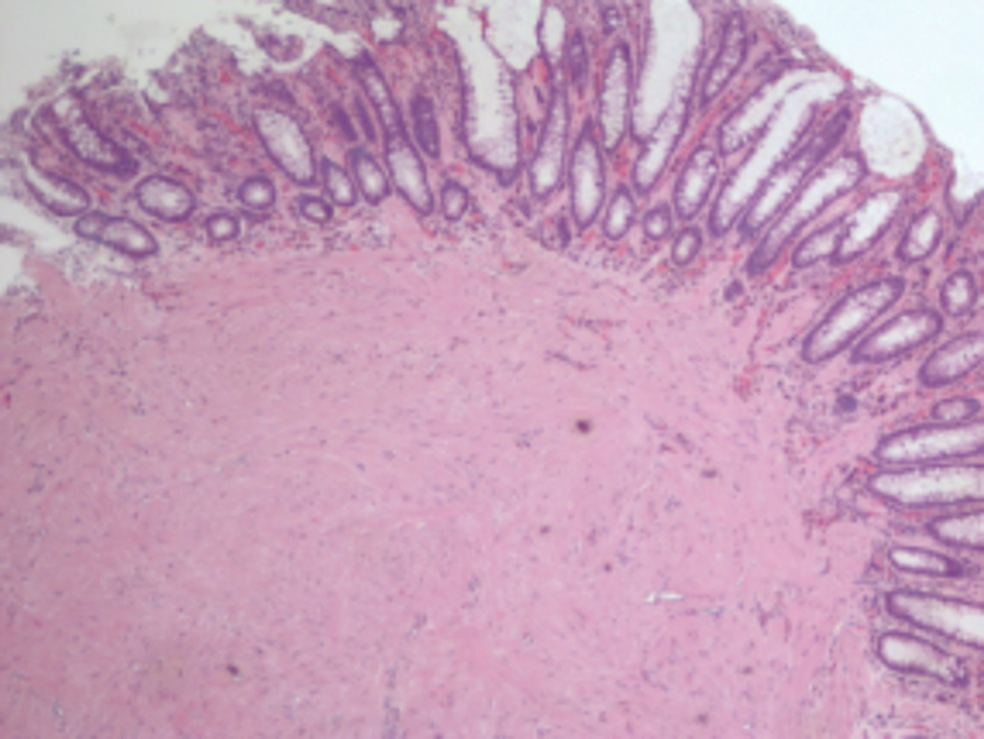
The image demonstrates a nodule composed of spindle cells with eosinophilic cytoplasm (40x)

**Figure 5:**
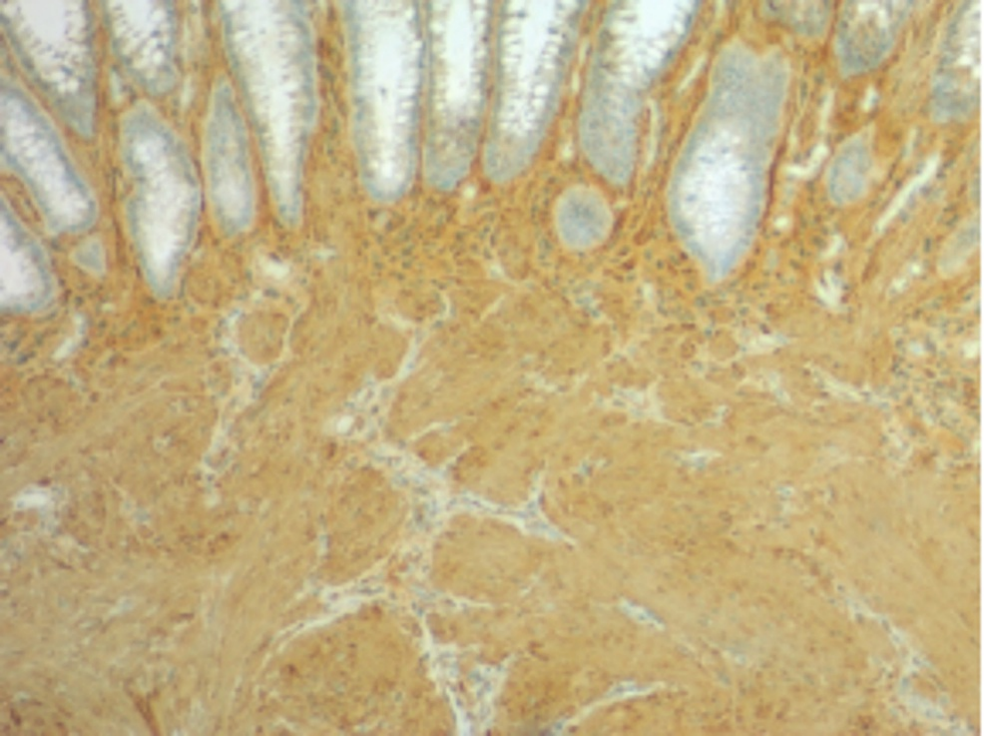
The image demonstrates that spindle cells are positive for SMA Biopsy also showed that spindle cells were negative for CD117 and CD34, indicating smooth muscle origin and compatibility with a submucosal leiomyoma. SMA: smooth muscle actin

A 59-year-old Hispanic female with a medical history of hypothyroidism, dyslipidemia, moderate persistent asthma, gastroesophageal reflux disease (GERD), constipation, colonic polyps presented to the gastroenterology clinic for surveillance colonoscopy. Three years prior, the patient was found to have a 1.3cm serrated tubular adenoma in the sigmoid colon. At the time of presentation, the patient was endorsing GERD-like symptoms that had been improving with omeprazole. She denied any melena, hematochezia, weight changes, weakness, and changes in stool caliber. A repeat colonoscopy was performed, which showed a 7mm sessile polyp (Figure 1) in the transverse colon. Epinephrine (1:100,000) was injected into the base of the polyp until adequate blanching was obtained. Using the Erbe cautery device, the polyp was snared (Figure 2) and sent for histopathologic analysis (Figures 3, 4). Biopsy showed a submucosal microscopic leiomyoma (Figure 5). The patient was advised to follow up in five years for surveillance. Upon 5-year follow-up, the patient endorsed no complaints. Repeat surveillance colonoscopy showed diverticulosis in the descending colon and internal hemorrhoids in rectum. 

## Discussion

A study by Miettinen et al., which evaluated 88 tumors of the muscularis mucosae of the colon and rectum, exhibited that these tumors have a significantly higher incidence in males with a median age of 62 years and are located predominantly in the rectum and sigmoid colon [1]. Upon histochemical evaluation of the lesions, they are positive for smooth muscle actin and desmin but negative for CD34, CD117, or S100 protein [1]. These are important findings as leiomyomas of the GI tract must be differentiated from gastrointestinal stromal tumors (GIST) due to their similar appearance under light microscopy. 

There is little known about the genetics of colonic leiomyomas; however, in some esophageal leiomyomas, deletions in collagen type IV alpha 5 and alpha 6 genes (COL4A5/COL4A6) on the X-chromosome have been identified and may be tied to the proliferation of smooth muscle in these cases [4]. This may also explain why there is a stronger incidence in males.

Clinical manifestations of colonic leiomyoma are rare, as it is most often found incidentally on routine colonoscopy screening with performed polypectomy. However, they can produce bulk symptoms such as abdominal pain, constipation, bleeding, or luminal obstruction if it grows too large or develops in specific locations [5]. Leiomyomas larger than 2cm are more likely to cause bulk-related symptoms, altered bowel habits, a palpable abdominal mass, and/or bloody stools [6]. Our patient exhibited a history of constipation and improving GERD-like symptoms. Small leiomyomas (less than 1cm) can rarely cause iron deficiency anemia [6]. Rectal leiomyomas specifically can produce additional symptoms of melena or anal bleeding [7]. Rarely, rectal and sigmoid leiomyomas can mimic gynecologic tumors if extension into the pelvis occurs. In women, this can present as a solid adnexal mass [8]. Symptomatic rectal leiomyomas have also been reported in children as young as 10 months [9].

Although colonic leiomyomas seldom have malignant potential, tumors containing high mitotic count, cellular atypia, and large size should be suspicious for possible metastasis [5]. If significant atypia is found in muscularis mucosa leiomyomas, they are sometimes referred to as “symplastic leiomyomas” [1]. This is differentiated from leiomyosarcoma, which is a larger, more aggressive tumor of the muscularis layer with copious mitoses and significant malignant potential. Histologically, the distinction with leiomyosarcomas is challenging and primarily based on the presence of necrosis, nuclear pleomorphism, cellularity, tumor size, and the number of mitotic figures [3]. Imaging findings and preoperative biopsy can be non-specific; thus, a combination of endoscopy with imaging studies such as CT scan, magnetic resonance imaging, and/or endoscopic ultrasonography firmly establishes the diagnosis [3]. 

On endoscopy, leiomyomas of the colon can look like ordinary mucosal adenomas, thus requiring biopsy and histological analysis to confirm the diagnosis [5]. Small sessile polyps have been reported as the most common appearance in many case reviews [1]; however, there have also been reports of leiomyomas of the colon appearing predominantly as firm, well-circumscribed, intraluminal, semi-pedunculated, or pedunculated polyps [10]. Because of their similar appearance to mucosal polyps, colonic leiomyomas are often misdiagnosed. Choi et al. reported that only about 46% of colorectal leiomyomas were diagnosed accurately based on their endoscopic features [6]. Ikeda et al. speculated this could be due to the scarcity of leiomyomas occurring as pedunculated lesions; thus, physicians are less likely to identify them morphologically [11]. However, the rate of accurate diagnosis of colorectal leiomyoma has been increasing with the increased frequency of screening colonoscopies [6].

A wide array of operative methods have been used to treat colonic leiomyomas, from simple endoscopic excision for small leiomyomas to subtotal colectomy for extensive, larger ones. The treatment of choice for most colonic leiomyomas is surgical excision [5]. Most patients require surgery to ensure accurate diagnosis and adequate removal. However, histological analysis combined with the location, tumor size, and mitotic count should be evaluated prior to determining the most effective treatment approach and/or the extent of resection [3]. This can paint a better picture of the grade and prognosis of the tumor. Due to the difficulty in differentiating benign vs. malignant tumors perioperatively, wide resection is recommended for smooth muscle tumors of the GI tract [5]. Surgical resection should be done for large pedunculated or sessile leiomyomas, while colonoscopic removal can be done for small pedunculated polyps [5]. There have only been a few reported cases of endoscopic removal of leiomyoma of the colon; these have been small (less than 2cm in diameter) [12]. However, Lee et al. successfully removed a 4.5cm intraluminal colonic leiomyoma via endoscopic polypectomy, proving that endoscopic resection of colonic leiomyoma is a cost-effective and less-invasive alternative to surgical resection [12].

Tumor size appears to be the most accurate prognostic predictor for colorectal smooth-muscle tumors [10]. Tumor size greater than 5cm suggests malignancy [6]. However, there is no evidence of colonic leiomyoma malignant transformation into leiomyosarcoma [6]. The overall prognosis of colonic leiomyoma is good, with no recurrence after resection documented in literature [3]. A study done by Choi et al. suggests that small polypoid leiomyomas (less than 2cm) originating from the muscularis mucosae can be successfully treated with endoscopic resection [6]. Furthermore, lesions less than 5mm can be extracted using cold biopsy forceps without tumor recurrence [6]. Endoscopic ultrasound can guide decision-making when considering endoscopic removal, as it can provide information on the size and extension of the tumor [6]. If any properties of the tumor suggest malignancy, such as the size of the leiomyoma being greater than 5cm, surgical resection is indicated [6,12]. For larger lesions, wedge colon resection may be necessary to secure complete removal [3]. 

## Conclusions

This rare case presentation strengthens the notion that endoscopic polypectomy may be superior to the current treatment choice for removal of most colonic leiomyomas, which is surgical excision. It also encourages further investigation towards the approach to remove colonic leiomyomas. Current practice guidelines recommend surgical intervention for colonic leiomyomas measuring more than 5cm due to the potential for malignant transformation. In this case, a leiomyoma in the transverse colon was removed via colonic polypectomy, which ultimately provided a less-invasive and less-costly procedure without complications or recurrence after five years.
